# Supplementary material for: The gender gap in STEM: (Female) teenagers’ ICT skills and subsequent career paths
Source: PLoS One. 2025 Jan 16;20(1):e0308074. doi: 10.1371/journal.pone.0308074 (PMC11737668; doi:10.1371/journal.pone.0308074)
Supplement: S1 Table — (ZIP) [file pone.0308074.s001.zip › S1_Table.pdf]

1 S1 Table.

Table 1. Comparison of sample with full data.

	Sample		Full data		Comparison	
	N	Mean	N	Mean	Diff.	p-value
ICT skills in 9th grade	9,315	0.569	14,486	0.552	0.017	0.000
Female	9,315	0.501	16,319	0.495	0.006	0.318
Migration Background	9,315	0.219	16,159	0.263	-0.044	0.000
Parent in STEM Occupation	9,315	0.337	14,912	0.305	0.032	0.000
Mathematical Skills	9,315	0.499	14,523	0.459	0.040	0.000
Choose STEM: longest training	9,315	0.326	11,016	0.320	0.006	0.381
Choose STEM: first training	9,315	0.337	11,001	0.331	0.006	0.355
Choose STEM: last training	9,315	0.317	10,966	0.313	0.005	0.488

The table summarizes the differences between the means of the variables of interest in the full data and the sample we selected. We restricted the sample to students for which data on competence tests, on socio-demografic characteristics, and their educational decisions are available.
