# Supplementary material for: The gender gap in STEM: (Female) teenagers’ ICT skills and subsequent career paths
Source: PLoS One. 2025 Jan 16;20(1):e0308074. doi: 10.1371/journal.pone.0308074 (PMC11737668; doi:10.1371/journal.pone.0308074)
Supplement: S2 Table — (ZIP) [file pone.0308074.s002.zip › S2_Table.pdf]

1 S2 Table.

Table 1. Comparison of sample with full data.

Statistic	N	Mean
ICT skills in 12th grade	2,789	0.299
Female	2,789	0.564
Migration Background	2,789	0.170
Parent in STEM Occupation	2,789	0.343
Mathematical Skills	2,789	0.619
Choose STEM: longest training	2,789	0.332
Choose STEM: first training	2,789	0.345
Choose STEM: last training	2,789	0.320

The table provides the mean of variables of interest. These are based on the sample of 2,789 students who participated in a second test on digital skills when attending 12th grade of high school.
