# Supplementary material for: The gender gap in STEM: (Female) teenagers’ ICT skills and subsequent career paths
Source: PLoS One. 2025 Jan 16;20(1):e0308074. doi: 10.1371/journal.pone.0308074 (PMC11737668; doi:10.1371/journal.pone.0308074)
Supplement: S3 Table — (ZIP) [file pone.0308074.s003.zip › S3_Table.pdf]

# 1 S3 Table.

**Table 1. Alternative definitions of outcome in main regression.**

| | *Dependent variable:* *Respondent chooses STEM occupation after high school* | | |
|---|---|---|---|
| | Longest training | First training | Last training |
| | (1) | (2) | (3) |
| ICT skills | −0.016 | 0.009 | −0.011 |
| | (0.041) | (0.041) | (0.041) |
| ICT skills * Female | 0.194*** | 0.176*** | 0.165*** |
| | (0.059) | (0.059) | (0.059) |
| Female | −0.496*** | −0.483*** | −0.481*** |
| | (0.031) | (0.031) | (0.031) |
| Migration Background | −0.008 | −0.002 | −0.005 |
| | (0.011) | (0.011) | (0.011) |
| Parent(s) in STEM occupation | 0.074*** | 0.074*** | 0.071*** |
| | (0.009) | (0.009) | (0.009) |
| Mathematical Skills | 0.038*** | 0.042*** | 0.029* |
| | (0.015) | (0.015) | (0.015) |
| Mathematical Skills * Female | 0.043** | 0.027 | 0.056*** |
| | (0.021) | (0.021) | (0.021) |
| Observations | 9,315 | 9,315 | 9,315 |
| $R^2$ | 0.174 | 0.174 | 0.171 |
| Adjusted $R^2$ | 0.173 | 0.173 | 0.170 |

The dependent variable is a binary variable that equals 1 if the respondents' *longest* training period within five years after completing secondary schooling was within a STEM field (column 1), if the respondents' *first* training period after completing secondary schooling was within a STEM field (column 2) or if the respondents' *last* training period within five years after completing secondary schooling was within a STEM field (column 3). All regressions also include a constant. Robust standard errors are clustered on the levels of schools. Significance: *p<0.1; **p<0.05; ***p<0.01.
