# Supplementary material for: The gender gap in STEM: (Female) teenagers’ ICT skills and subsequent career paths
Source: PLoS One. 2025 Jan 16;20(1):e0308074. doi: 10.1371/journal.pone.0308074 (PMC11737668; doi:10.1371/journal.pone.0308074)
Supplement: S4 Table — (ZIP) [file pone.0308074.s004.zip › S4_Table.pdf]

1 S4 Table.

Table 1. ICT skills in 9th and 12th grade and career paths: Direct relationship and moderating effect (using WLE scores)

	Dependent variable: Respondent chooses STEM occupation after high school							
	**ICT skills in 9th grade**						**ICT sk**	
	(1a)	(1b)	(1c)	(1d)	(1e)	(1f)	(2a)	(2
ICT skills	0.043***	-	0.042***	0.027***	0.015**	0.024***	0.086***	0.09
	(0.005)	-	(0.005)	(0.007)	(0.007)	(0.008)	(0.012)	(0.0
Female	-	−0.373***	−0.371***	−0.374***	−0.369***	−0.400***	−0.251***	−0.2
	-	(0.009)	(0.009)	(0.009)	(0.009)	(0.014)	(0.017)	(0.0
ICT skills * Female	-	-	-	0.033***	0.032***	0.013	-	−0.
	-	-	-	(0.009)	(0.009)	(0.011)	-	(0.0
Migration Background	-	-	−0.010	−0.009	−0.006	−0.006	−0.016	−0.
	-	-	(0.011)	(0.011)	(0.011)	(0.011)	(0.023)	(0.0
Parent(s) in STEM	-	-	0.075***	0.075***	0.073***	0.074***	0.072***	0.07
	-	-	(0.009)	(0.009)	(0.009)	(0.009)	(0.018)	(0.0
Mathematical Skills	-	-	-	-	0.043***	0.009	-	
	-	-	-	-	(0.011)	(0.015)	-	
Mathematical Skills * Female	-	-	-	-	-	0.066***	-	
	-	-	-	-	-	(0.021)	-	
Constant	0.322***	0.513***	0.486***	0.487***	0.463***	0.481***	0.426***	0.42
	(0.005)	(0.006)	(0.007)	(0.007)	(0.009)	(0.011)	(0.016)	(0.0
Observations	9,315	9,315	9,315	9,315	9,315	9,315	2,789	2,7
R^2	0.007	0.158	0.172	0.173	0.174	0.175	0.108	0.1
Adjusted R^2	0.007	0.158	0.171	0.172	0.174	0.174	0.106	0.1

The dependent variable is a binary variable that equals 1 if the respondents' *longest* training period within five years after completing secondary schooling was within a STEM field. The ICT skills relate to the tests conducted in 9th grade (columns 1a to 1f) and 12th grade (columns 2a to 2d). In contrast to the results in Table **??**, we now use the WLE score provided in the NEPS data to measure the ICT skills. Due to panel attrition, the subset of students who were again tested in 12th grade was much smaller. In columns 2a to 2d we only consider the selection into a STEM field after grade 12. Robust standard errors are clustered on the levels of schools. Significance: *p<0.1; **p<0.05; ***p<0.01.
