# Supplementary figures and images for: The gender gap in STEM: (Female) teenagers’ ICT skills and subsequent career paths
Source: PLoS One. 2025 Jan 16;20(1):e0308074. doi: 10.1371/journal.pone.0308074 (PMC11737668; doi:10.1371/journal.pone.0308074)

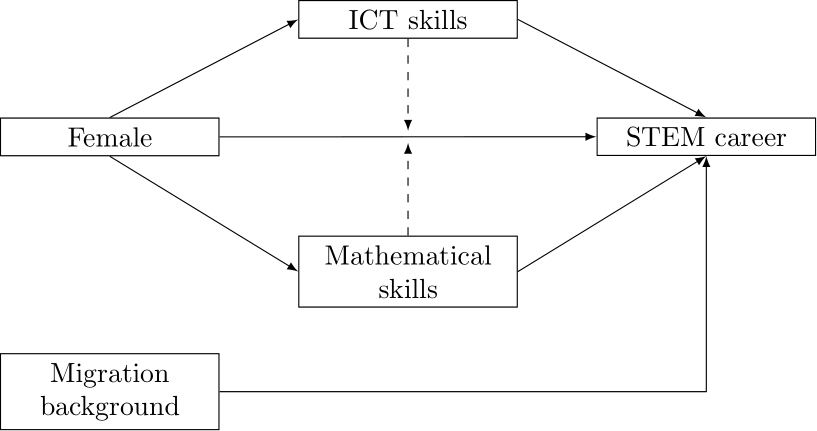

Supplement: S1 Fig — illustrates the empirical analysis including all variables. In addition to ICT skills and gender, we also include a teenager’s migration background as well as their mathematical skills. As we do not know a priori whether ICT (or mathematical) skills moderate the gender-specific selection into STEM, we also include interaction terms in some specifications as indicated by the dashed arrow. Please note that Fig 2 in the main text has concentrated on the main variables of interest—gender, ICT skills, and choice of a STEM career. (PNG) [file pone.0308074.s005.png]
